# Validation of polyester nasal swabs for post-mortem SARS-CoV-2 diagnosis in Karachi, Pakistan: a prospective surveillance analysis

**DOI:** 10.7189/jogh.15.04288

**Published:** 2025-11-21

**Authors:** Raheel Allana, Fatima Aziz, Sameer Mohiuddin Belgaumi, Furqan Kabir, Inci Yildirim, Aneeta Hotwani, Fauzia Aman Malik, Obianuju Aguolu, Sahrish Muneer, Nazia Ahsan, Zahra Hasan, Saad B Omer, Abdul Momin Kazi

**Affiliations:** 1 Department of Paediatrics, Aga Khan University, Karachi, Pakistan; 2 Peter O’ Donnell Jr School of Public Health, UT Southwestern Medical Centre, Texas, USA; 3 Department of Pediatric Infectious Diseases, Yale School of Medicine, New Haven, USA; 4 Division of Epidemiology, Public Health Department, Ohio State University, Columbus, USA; * Joint first authorship.

## Abstract

**Background:**

The COVID-19 pandemic has significantly impacted global health, with low- and middle-income countries (LMICs) facing unique healthcare challenges. Polyester nasal swabs stored in dry tubes have emerged as a cost-effective and scalable method for routine testing of severe acute respiratory syndrome coronavirus 2 (SARS-CoV-2). We aimed to assess the prevalence of SARS-CoV-2 among deceased individuals in an urban slum in Karachi, Pakistan, using dry and wet polyester nasal swabs, and to validate their use for post-mortem detection of the virus.

**Methods:**

We conducted a prospective observational study from July 2022 to August 2023 in a low-income setting. We collected nasal samples from 350 deceased individuals based on community death alerts using dry polyester and wet swabs with transport media. These were then processed for SARS-CoV-2 using reverse transcription polymerase chain reaction (RT-PCR), with the positive samples sequenced on the Illumina platform to identify circulating variants. We also performed a comparative analysis between dry and wet swab methods for diagnostic performance.

**Results:**

Of the 350 samples, 21 (6.0%) tested positive for SARS-CoV-2. Males accounted for 15/21 (71.4%) of positive cases, with the majority aged 60 and above (n/N = 12/21, 57.1%). The Omicron (22F) variant was the most prevalent, detected in 16/21 (76%) cases. The diagnostic performance of wet swabs showed a sensitivity of 76.19%, while dry swabs were more accurate, with a sensitivity of 90.48%, achieving a diagnostic odds ratio of 3120.5.

**Conclusions:**

Our study demonstrated the feasibility and effectiveness of using dry polyester nasal swabs for post-mortem detection of SARS-CoV-2 in resource-constrained settings. These findings emphasise the method's potential for monitoring respiratory infectious diseases and guiding public health strategies in LMICs.

The COVID-19 pandemic, caused by severe acute respiratory syndrome coronavirus 2 (SARS-CoV-2), posed a serious challenge to public health globally [1]. As countries grappled with the multifaceted impact of the virus, understanding its toll on mortality rates became a crucial goal for research efforts worldwide. Many surveys were conducted during the pandemic to assess the prevalence of SARS-CoV-2 [2,3]. Serial population-based serosurveys in two peri-urban areas of Karachi, conducted in April, June, and August 2020, provided insights into the infection fatality rate, reporting 1.7%, 0.4%, and 0.3% during phases 1, 2, and 3, of the pandemic, respectively [4]. Such data are essential for shaping public health strategies and allocating resources in the face of evolving pandemics. These findings also highlighted how temporal variations in community transmission and public health interventions might influence the observed infection fatality rate, emphasising the dynamic nature of pandemic surveillance. However, a better understanding of the full impact of SARS-CoV-2 could be achieved through robust methods for post-mortem diagnosis, particularly in low- and middle-income countries (LMICs), where resource constraints pose additional challenges. In these contexts, identifying cost-efficient, scalable, and logistically feasible diagnostic tools is critical for maintaining continuous surveillance systems, especially where healthcare systems are overwhelmed.

Some testing methods for widespread SARS-CoV-2 detection have been challenging to implement due to feasibility and cost-related barriers. For example, upper respiratory swabs are traditionally transferred to the laboratory in viral transport media (VTM), which preserves viral RNA and maintains the its viability during transport [5]. However, the sudden onset of the SARS-CoV-2 pandemic led to a surge in global testing demands and significant shortages of VTM. This prompted laboratories and healthcare systems to explore alternative VTM and collection strategies, including the use of saline, phosphate-buffered saline (PBS), and dry swabs to maintain diagnostic testing capacity despite supply chain constraints [6].

Amid these challenges, dry storing polyester nasal swabs in collection tubes emerged as a practical and cost-effective approach for self-collection [7], especially as the method is easy to use, lowers cost, and is independent from cold-chain requirements [8]. Although traditionally not favoured because they lack a liquid medium to stabilise viral particles, studies have demonstrated that dry swabs can yield comparable diagnostic accuracy for molecular detection when processed promptly or stored appropriately [9]. Their utility has been especially significant in resource-limited settings and during periods of high testing demand, helping to maintain testing throughput despite logistical challenges. This innovative approach not only addresses logistical challenges but also ensures the reliability and robustness needed for effective testing, thereby presenting a promising solution for scalable and accessible diagnostic efforts [10].

Gokulan and colleagues demonstrated that dry swabs, when processed without RNA extraction, maintain high diagnostic sensitivity for SARS-CoV-2 detection and show comparable or even lower cycle threshold values than samples in VTM [11]. Khan and colleagues compared a kit-independent method of detecting SARS-CoV-2 RNA using dry swabs with standard kit-based RNA isolation techniques. They reported a sensitivity of 94.64% in the 56 samples tested, indicating that dry swabs can be highly effective for detecting SARS-CoV-2 [12]. However, direct comparative evidence between dry and wet nasal swabs post-mortem, remains limited, and such data would help to guide protocol standardisation for surveillance systems. This is especially true for post-mortem surveillance, where further research would help validate both the reproducibility and accuracy of the polyester dry swab method, establishing its clinical applicability.

Importantly, integrating such diagnostic methods into community-based mortality surveillance frameworks could enhance early detection of respiratory disease outbreaks, improve pathogen-specific mortality data, and inform timely public health interventions. This would ensure the stringent standards necessary for the effective and reliable polyester swab approach for SARS-CoV-2, contributing to enhanced diagnostic procedures and public health measures. In this study, we assessed the feasibility and validity of dry *vs.* wet polyester nasal swabs for SARS-CoV-2 detection among deceased individuals.

## METHODS

### Study design and setting

We conducted a prospective, observational SARS-CoV-2 surveillance and method validation study focussing on deceased members of the urban slum population of Ali Akbar Shah in Karachi, Pakistan. The area, which has an approximate population of 249 128, is covered by the Aga Khan University's Health and Demographic Surveillance System, which includes primary healthcare centres [13]. This study was a part of the broader ‛COVID-19, Burial Site Surveillance to Measure Excess Mortality in Pakistan’ project, and spanned 13 months, from September 2022 to October 2023. The study was approved by the ethics review committee of the Aga Khan University (2021-6445-19025).

### Study participants and identification

Eligible participants were recently deceased community members, whose next-to-kin, after being informed on study procedures, provided consent on their behalf for post-mortem swab collection. Next-of-kin refers to individuals closely related to the deceased, such as parents, primary caregivers, spouses, or other immediate family members. Since this was a community-based study, the identification for sample collection relied on death alerts from various community sources, including local community health workers, council members, religious leaders, and others.

### Sampling strategy

**Figure 1:**
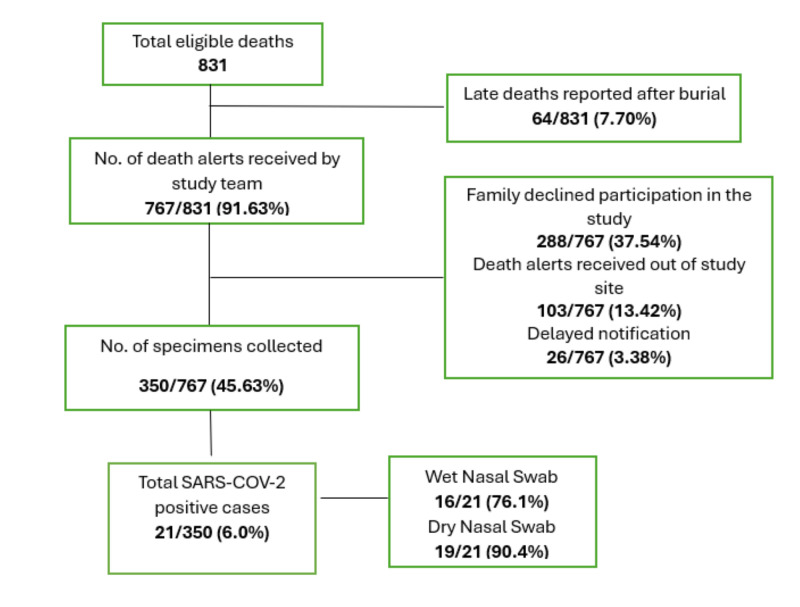
Sampling strategy for the nasal swab collection process.

We collected 30–50 nasal samples per month from deceased community members in consenting households, in response to death alerts received from community sources. This process continued until we reached a total sample size of 350 individuals. We tested all samples against SARS-CoV-2 using reverse transcription polymerase chain reaction (RT-PCR) (**Figure 1**).

### Religious, contextual and procedural considerations

The study’s sampling method was deemed religiously acceptable because of its close alignment with Muslim ritual washing of the deceased body, which includes the cleaning of the nostrils with cotton swabs. This was confirmed through focus group discussions, religious scholar consultations, and a *fatwa* (Islamic religious edict) and similar religious rulings supporting post-mortem swab collection across Islamic, Christian, and Hindu communities. Death alerts were facilitated through larger community engagement and advocacy with religious leaders, traditional birth attendants, community members, community health workers, and funeral workers.

### Sample procedure and storage

Upon receiving a death alert, we visited the identified household, obtained informed verbal consent, completed a case record form, and performed the nasal swab collection from the deceased individual. For the wet swab with VTM, we collected a nasopharyngeal swab using a polyester-tipped swab with a plastic shaft, following standard procedures. For the dry swab, we used a single polyester swab with a plastic shaft to collect samples from both anterior nares (left and right) [14], inserting the swab into the nostril and ensuring contact with the nasopharynx to absorb secretions. While collecting and handling samples, we followed the standard operating procedure by wearing personal protective equipment. We collected post-mortem swab samples within a median of four hours after death (IQR = 3, 6), based on the time of death reported by the family during death alerts, transporting them to the laboratory in cold chain conditions and processing them within a median of 24 hours (IQR = 20, 30) of collection. We excluded samples arriving at the laboratory beyond this timeframe to minimise degradation risk. While viral RNA may degrade over time post-mortem, all efforts were made to minimise this variability by standardising the collection window.

### VTM evaluation: dry *vs.* wet nasal swabs

We used two different approaches to compare sampling methods:

- Dry nasal swabs: we collected nasal swabs without placing them in a liquid medium at the time of collection. We transported these dry swabs and later rehydrated them in the laboratory by adding 2.5 mL of PBS and incubating them for 10 minutes before RNA extraction for further molecular testing.
- Wet nasal swabs in VTM: we placed swabs directly into VTM at the time of collection, which serves as a liquid preservative medium.

### Verbal autopsy and grief support

One week after sample collection, we conducted verbal autopsies using the World Health Organization (WHO) 2016 verbal autopsy (VA) tool, supplemented with SARS-CoV-2 items. Two independent physicians assigned probable causes of death, while a third adjudicated discrepancies. A psychologist also provided grief support during interviews and offered regular counselling to the study team to manage emotional strain. We also reported the results related to the cause of death separately [15].

### Laboratory analysis

Following collection, we transported the dry and wet swabs in a temperature-controlled cooler at 2–8°C to the Infectious Diseases Research Laboratory at the Aga Khan University for further processing. We stored samples collected from both standard universal transport medium (UTM) and dry nasal swabs at −80°C before being processed.

We tested samples for the presence of SARS-CoV-2 RNA using RT-PCR. We performed dry swab processing by adding 2.5 mL of PBS, followed by vortexing for 30 seconds and incubating for 10 minutes before RNA extraction, which, in turn, was done using QIAamp viral RNA mini kit (Qiagen, Hilden, Germany) according to the manufacturer's instructions and then sent to the clinical laboratory for SARS-CoV-2 detection using the FDA-approved COBAS® SARS-CoV-2 RT-PCR assay. Further, we measured the cycle threshold (CT) values using quantitative PCR to assess viral loads, with mean (x̄) values and standard deviations (SD) calculated to reflect variability between replicates. We considered a sample positive for SARS-CoV-2 if the RT-PCR CT value was ≤35 for at least one target gene, based on the COBAS® assay protocol. We compared the samples based on their CT values, which indicate viral load levels, and we analysed the results for consistency and variability.

### SARS-CoV-2 genome sequencing

We subjected viral RNA from SARS-CoV-2 RT-PCR-positive samples to amplicon-based genomic sequencing using the Illumina sequencing platform [16]. In parallel, we prepared the libraries for all samples using the Illumina COVIDSeq Assay (containing 21 samples) with the V4.1 primers pool according to the ARTIC Lo-Cost protocol using the Illumina iSeq100 platform [17]. We carried out no template control reactions to confirm the validity of positive ones, as carry-over products can disrupt sequencing accuracy, especially when dealing with low-copy targets. We used the IDseq pipeline to generate the consensus genome, and we uploaded raw sequencing data in FASTQ format to the Chan Zuckerberg ID platform [18] for automated bioinformatic processing. We selected the SARS-CoV-2 consensus genome pipeline, which performs a series of standardised analytical steps, including quality control and adapter trimming, alignment of sequencing reads to the SARS-CoV-2 reference genome (NC_045512.2), consensus genome assembly, variant detection, and calculation of genome coverage and identity metrics. For each sample, we extracted the following sequencing quality parameters from the Chan Zuckerberg ID platform output reports: percentage identity to the reference genome, total number of informative (non-N) bases, number of missing bases (n) and ambiguous bases (represented by International Union of Pure and Applied Chemistry codes) in the consensus sequence, mean depth of coverage ( × ), breadth of genome coverage (%), and the total number of single nucleotide polymorphisms (SNPs) relative to the reference. We used these metrics to compare sequencing performance between dry and wet nasal swab groups [19].

### Data analysis

We used descriptive statistics to present categorical and continuous variables. We calculated inferential tests, such as sensitivity, specificity, positive predictive value (PPV), negative predictive value (NPV), and diagnostic odds ratio (dOR), to evaluate the diagnostic accuracy of the tests. We calculated receiver operating characteristic (ROC) curves and area under the curve (AUC) values to assess overall test performance using STATA, version 18 (StataCorp LLC, College Station, TX, USA). We performed comparative analysis of genomic sequencing data to identify the prevalence and variability of SARS-CoV-2 variants.

## RESULTS

We analysed 350 cases, with 21 individuals testing positive (6.0%) and 329 testing negative (94.0%) for SARS-CoV-2. By gender, 71.42% of positive cases were male, while 28.57% were female. Based on data from death alerts, most deaths occurred among individuals aged 60 years and older (57.14%). Regarding location, 61.1% deaths occurred at home, 33.1% in hospitals, and 5.7% on the way to a healthcare facility.

Dry nasal swab results showed 90.4% positive cases, while wet nasal swab results showed 76.19% (**Table 1**). Specifically, there were 14 occurrences of 22F, six of 22B, and a handful of other variants spread across the sample sets (**Table 2**). The genomic sequencing of SARS-CoV-2 variants revealed a variety of Omicron subvariants and recombinant strains across two sample groups, dry (n = 19) and wet (n = 16). The 22F was the most prevalent Omicron subvariant overall and in both swab subgroups, with nine occurrences in the wet swab and seven in the dry swab group. We also identified three cases of the 22B Omicron variant in both groups, as well as recombinant variants in small numbers, with three cases in dry and two in wet swabs. Other Omicron variants, like 21K and 22E, appeared with varying frequencies across the two groups, with one or two cases in each.

**Table 1:** SARS-CoV-2 status and demographic distribution among deceased individuals

	Positive SARS-CoV-2 status (n = 21, 6.0%), n (%)	
	**Dry**	**Wet (UTM)**
**Gender**		
Male	14 (66.6)	12 (57.1)
Female	5 (19.0)	4 (19.0)
**Age at death in years**		
Under 5	3 (14.2)	2 (9.5)
5–9	1 (0.0)	1 (0.0)
Above 18	14 (66.6)	13 (61.9)
**Place of death**		
Home	9 (42.8)	8 (38.1)
Hospital	7 (33.3)	7 (33.3)
On route to hospital	2 (9.5)	1 (4.7)
**Respiratory symptoms**		
Presence of RTI	5 (28.5)	2 (9.5)
Severe symptoms	1 (4.7)	1 (4.7)
Very severe	4 (19.0)	3 (14.2)
No respiratory symptoms	12 (57.1)	12 (57.1)

RTI – respiratory tract infection, SARS-CoV-2 – severe acute respiratory syndrome coronavirus 2, UTM – universal transport media

**Table 2:** Genomic sequencing of SARS-CoV-2 variants

Genome name	Dry (n = 19)	Wet (n = 16)
22F	7	9
22B Omicron	3	3
Recombinant	3	2
23A	1	1
XEC recombinant	1	0
21K	1	1
22E	1	0
21L	1	0
Unable to align	1	0

### Assessing the validity of nasal swabs

#### Dry nasal swabs

The diagnostic performance metrics for the RT-PCR test using dry nasal swabs showed a sensitivity of 90.48% (95% CI = 69.6, 98.8) with 19/21 cases detected, specificity of 100% (95% CI = 98.9, 100), PPV of 100% (95% CI = 82.4, 100), NPV of 99.39% (95% CI = 97.8, 99.9%), and a dOR of approximately 3120.5 (**Table 3**, Panel A).

**Table 3:** Assessment of dry and wet nasal swabs

	RT-PCR positive, n	RT-PCR negative, n	Total	Metrics
**Standard dry swab positive**	19	0	19	PPV = 100%
**Standard dry swab negative**	2	329	331	NPV = 99.39%
**Total**	21	329	350	
	Sensitivity = 90.48%	Specificity = 100%		dOR = 3120.5
**Standard wet swab positive**	16	0	16	PPV = 100%
**Standard wet swab negative**	5	329	334	NPV = 98.43%
**Total**	21	329	350	
	Sensitivity = 76.19%	Specificity = 100%		dOR = 1049.6

dOR – diagnostic odds ratio, NPV – negative predictive value, PPV – positive predictive value

#### Wet nasal swabs

The diagnostic performance metrics for the RT-PCR test using wet nasal swabs showed a sensitivity of 76.19% (95% CI = 52.8, 91.8) with 16/21 cases detected, specificity of 100% (95% CI = 98.9, 100), PPV of 100% (95% CI = 79.4, 100), NPV of 98.43% (95% CI = 96.5, 99.5), and a dOR of approximately 1049.6 (**Table 3**, Panel B).

**Figure 2:**
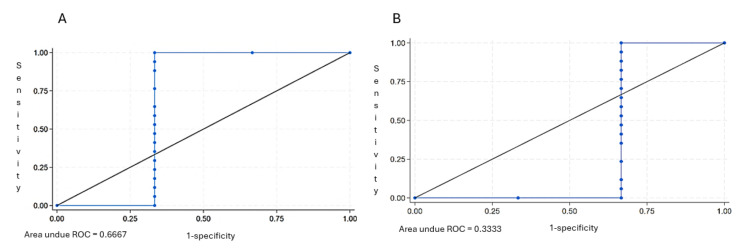
**Panel A.** ROC curve for the Dry Nasal Swab CT values in predicting PCR-positive results. **Panel B.** ROC curve for the Wet Nasa Swab CT values in predicting PCR-positive results.

We further performed ROC curve analysis to evaluate diagnostic performance. The AUC was 0.667 for dry swabs and 0.33 for wet swabs, indicating a difference in diagnostic performance. Dry swabs consistently demonstrated better sensitivity across thresholds, supporting their use in post-mortem SARS-CoV-2 testing where reliable detection is critical (**Figure 2**, Panels A and B).

**Figure 3:**
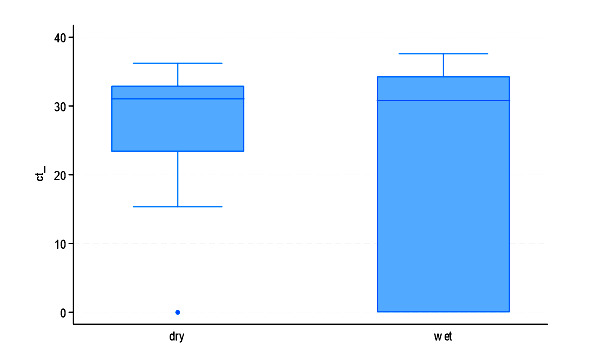
Distribution of CT values from dry and wet nasal swabs among SARS-CoV-2 positive post-mortem cases (n = 21). Each boxplot shows the IQR, median (line within the box), and whiskers representing the full range excluding outliers.

Further, the comparison of dry nasal swabs and wet swabs revealed notable differences in sequencing quality and genomic coverage. The average CT values were comparable between the two groups, with slightly lower results for dry nasal swabs (29.1) than wet swabs (31.3). A few outliers were visible with higher CTs in wet swabs (**Figure 3**). The visual spread suggests consistent detection performance, though dry swabs may have yielded slightly lower CT values overall in some cases. The percentage identification of the consensus genome with the reference genome was slightly higher in dry nasal swabs (99.7%) than in wet swabs (99.6%). Wet swabs showed a greater number of informative bases (n = 17 780) compared to dry nasal swabs (14 809), but the latter had more missing bases (n = 10 627) than the former (n = 9977). The average ambiguous bases were lower in dry nasal swabs (0.89) compared to wet swabs (2.8), suggesting better sequence clarity. The average depth coverage was higher in wet swabs (1304x) than in dry nasal swabs (1215x), and the breadth coverage was also greater in the former (63.3%) than the latter (54.25%). Additionally, the average number of SNPs detected was higher in wet swabs (56) compared to dry nasal swabs (46.1). These findings indicate that dry nasal swab samples had a higher viral load compared to wet swabs, providing better sequencing depth and coverage, potentially improving genome assembly and variant detection (Table S1 in the **Online Supplementary Document**).

## DISCUSSION

This study is one of a few to evaluate both dry and wet swab specimens from post-mortem cases across a wide age spectrum, including both adults and children, in a resource-constrained setting. The findings demonstrate that dry nasal swabbing using polyester materials exhibits a high sensitivity of 90.48% and perfect specificity of 100%, as compared to wet swabs, which showed a sensitivity of 76.19% and an equally high specificity. This not only affirms the reliability of polyester swabs for detecting SARS-CoV-2, but also indicates their feasibility for its accurate detection in deceased individuals, even in challenging environments where maintaining cold-chain storage might be difficult. By simultaneously administering and comparing both dry and wet swabs, we implemented a dual-modality sampling strategy, which is rarely reported in mortality surveillance literature. This comprehensive approach not only maximised diagnostic yield, but also enabled internal validation across specimen types, thereby enhancing the robustness and reliability of pathogen detection. The inclusion of a broad age range and multiple specimen types enhance our study's relevance for post-mortem pathogen detection and informing pathogen-specific burden estimates in LMICs.

Our results are consistent with the study conducted by Leah and colleagues, which used dry nasal swabs for SARS-CoV-2 detection in both adult and paediatric populations. They found that polyester nasal swabs stored in dry collection tubes provided a reliable method for SARS-CoV-2 viral load testing, as viral RNA remains stable under conditions required for laboratory shipment [20]. Our findings also align with those of Antonios and colleagues [21], demonstrating that dry swabs deliver better diagnostic performance in rapid antigen testing for SARS-CoV-2. Moreover, the clinical comparison confirmed the in vitro experimentation, showing that the dry nasopharyngeal swab performed slightly better than the wet one (sensitivity increasing from 35% to 47%), likely due to a decreased dilution of the sample [21]. We also found that our dry nasal swabs were more sensitive than wet swabs transported in UTM.

Our results are in contrast with the study by Hart and colleagues, which evaluated the performance of nasal swabs in convalescent SARS-CoV-2 patients and reported a sensitivity of 75%. In comparison, our study demonstrated a significantly higher sensitivity of approximately 90.48% for dry nasal swabs in post-mortem samples from deceased individuals [22].

At the same time,e in a cross-sectional study conducted at the Hospital of Pennsylvania, Codrington and colleagues found no difference between dry and wet swabs for MRSA detection, suggesting that neither method offered a superior yield [23]. However, the authors noted that dry swabs require less preparation than wet ones, making them a more practical and preferable choice for MRSA screening. This highlights that, while dry swabs may not always show significantly higher sensitivity, their methodological advantages still support their use in clinical practice.

It is important to note that all of the above-mentioned research was conducted on living individuals. In contrast, we performed our study on post-mortem samples obtained from deceased individuals, where dry swabs continued to demonstrate superior or comparable sensitivity.

Lastly, even in other anatomical contexts, evidence suggests that dry swabs consistently demonstrate superior diagnostic performance [24,25]. For example, several studies have reported that dry swabs show better results compared to pre-moistened swabs for the detection of *group A streptococci via* throat swabs. This challenges the conventional assumption that moistening swabs prior to sample collection enhances sensitivity. Conversely, dry swabs may offer advantages by preserving sample integrity, minimising dilution, and reducing the risk of contamination, particularly in low-resource settings.

These findings [24,25] indicate that, in specific diagnostic settings such as streptococcal pharyngitis, dry swabs may provide a more reliable and accurate detection method [26]. Furthermore, Antonios and colleagues evaluated the diagnostic performance of dry *vs.* wet endocervical swabs for the detection of *Neisseria gonorrhoeae* infection offers valuable insights. They found that dry endocervical swabs had significantly higher sensitivity (96.3%) compared to wet swabs (91.5%), suggesting them to be more effective in capturing and preserving the target pathogen. This further strengthens the evidence that, irrespective of the anatomical context, whether detecting sexually transmitted infections from endocervical swabs or respiratory pathogens from nasopharyngeal swabs, dry swabs consistently outperform wet swabs in terms of sensitivity. The high specificity of both dry and wet polyester swabs observed in this study ensures minimal false positives, an essential feature for public health surveillance aimed at understanding the true burden of SARS-CoV-2 mortality. In settings where the health infrastructure is resource-constrained, the risk of overwhelming health systems with false positives could detract from pandemic response efforts [21]. Moreover, the genome sequencing component of the study, which identified predominant variants such as Omicron 22F and recombinant strains, emphasises the broader relevance of polyester nasal swabs beyond initial diagnostic testing. Identification of Omicron variants fits with the currently circulating strains identified in Pakistan during the study period [27]. The ability to sequence viral RNA from swab samples collected under such conditions shows the method's versatility. Comparable studies, such as genomic surveillance efforts in Brazil, have similarly utilised nasal swab samples to monitor variant emergence, though they primarily relied on nasopharyngeal swabs [28]. Thus, our study contributes to this global effort by validating polyester swabs as a feasible tool for genomic surveillance in LMICs.

### Strengths

Our study evaluated dry *vs.* wet nasopharyngeal swabs for pathogen detection, with a unique focus on post-mortem cases in an urban slum in Karachi, Pakistan. This population has been underrepresented in prior research, making the findings particularly significant for understanding local mortality patterns and the epidemiology of SARS-CoV-2 variants in resource-limited settings. One of the study’s primary contributions is its practical relevance to LMICs, where access to advanced diagnostic infrastructure is often limited. As dry swabs not only maintain diagnostic accuracy, but also outperform wet swabs in sensitivity, they can be an efficient, cost-effective alternative for pathogen detection. Their superior performance can likely be attributed to the avoidance of dilution that occurs with VTM in wet swabs, thereby preserving sample integrity, which is a critical factor in post-mortem diagnostics. Moreover, our study underscores the logistical feasibility of implementing dry swab collection in real-world, low-resource environments, supporting their broader adoption in similar contexts. These findings can therefore inform public health strategies and diagnostic protocols in LMICs, ultimately contributing to more effective disease surveillance and mortality analysis. Lastly, one of the advantages of the dry swab technique is its simplified logistics, *i.e.* eliminating the need for a liquid transport medium, which reduces reliance on VTM and simplifies sample collection. However, it is important to clarify that cold chain storage was still used during transport and laboratory processing in this study. Therefore, the logistical benefit of using dry swabs primarily lies in the absence of VTM, rather than full independence from cold chain infrastructure.

### Limitations

One of the main limitations of this study is the low number of SARS-CoV-2 positive samples (n = 21), limiting its statistical power and preventing us from drawing broader or firmer conclusions regarding the comparative diagnostic performance of dry *vs.* wet swab techniques, which should be done by larger studies across diverse settings. Further, we conducted the study in a specific urban slum population in Karachi, Pakistan, which may not be directly applicable to other regions with different healthcare infrastructures, cultural practices, or disease dynamics. This could affect the feasibility and effectiveness of using polyester swabs in other settings with different access to healthcare resources, diagnostic practices, and burial customs. Furthermore, although the sensitivity of dry swabs appeared higher than that of wet swabs, the overlapping 95% CIs suggest that this difference may not be statistically significant. In this context, the absence of formal hypothesis testing limits our ability to determine whether observed differences are due to chance. Additionally, factors such as the timing of sample collection post-mortem and environmental conditions (*e.g.* temperature, humidity) could influence the stability of samples and pathogen detection, which we did not fully explore. These variables could impact on the diagnostic accuracy of the swabs and should be considered in future studies. Expanding the scope of future studies to include alternative testing methods would provide a deeper understanding of the effectiveness of polyester swabs in different diagnostic contexts.

### Future directions

This study provides a foundation for future research on the use of polyester nasal swabs in broader respiratory disease surveillance. Given their high sensitivity and specificity, polyester swabs could be validated for other infectious diseases, such as influenza and RSV, which are prevalent in many LMICs. Expanding their use in surveillance programmes could enhance global preparedness by providing a diagnostic tool that is affordable, reliable, and scalable. Additionally, community-assisted collection methods for post-mortem diagnostics could be explored, reducing the burden on healthcare systems and improving the efficiency of mortality surveillance programmes. Incorporating next-generation sequencing or other advanced genomic methods could provide deeper insights into pathogen genomes, facilitating more accurate diagnostics and better monitoring of emerging strains.

## CONCLUSIONS

We found that polyester nasal swabs are a highly effective modality for post-mortem COVID-19 testing in resource-limited settings such as LMICs, where their high sensitivity and logistical advantages position them as an effective, scalable diagnostic option, especially for pandemic preparedness and response. Beyond its cost-effectiveness, this method helped maintain the stability of viral RNA under the diverse environmental conditions required for home collection and subsequent transportation to the laboratory. When compared to data from other countries, our findings highlight the role that simple, yet robust diagnostic methods can play a key role in enhancing public health surveillance, improving pandemic response efforts, and informing global strategies for managing infectious disease outbreaks. Despite the small sample size and the limitations mentioned above, these data offer valuable operational insights that can inform the development of national or WHO-endorsed protocols for post-mortem sample collection and SARS-CoV-2 detection.

## Additional material


Online Supplementary Document

## References

[1] Ukhurebor KE Singh KR Nayak V Gladys U-E Influence of the SARS-CoV-2 pandemic: a review from the climate change perspective. Environ Sci Process Impacts. 2021;23:1060–78. 10.1039/D1EM00154J 34132283

[2] Lai CC Wang JH Hsueh PR Population-based seroprevalence surveys of anti-SARS-CoV-2 antibody: An up-to-date review. Int J Infect Dis. 2020;101:314–22. 10.1016/j.ijid.2020.10.011 33045429 PMC7546669

[3] Shakiba M Nazemipour M Heidarzadeh A Mansournia M Prevalence of asymptomatic COVID-19 infection using a seroepidemiological survey. Epidemiol Infect. 2020;148:e300. 10.1017/S0950268820002745 33183367 PMC7783089

[4] Nisar MI Ansari N Khalid F Amin M Shahbaz H Hotwani A Serial population-based serosurveys for COVID-19 in two neighbourhoods of Karachi, Pakistan. Int J Infect Dis. 2021;106:176–82. 10.1016/j.ijid.2021.03.040 33737137 PMC8752032

[5] Locher K Velapatino B Caza M Li L Porter C Charles M Approach to assessment of new swabs and viral transport media for SARS-CoV-2 testing. J Clin Microbiol. 2021;59:01562–20. 10.1128/JCM.01562-20 33139423 PMC8091853

[6] Petersen J Dalal S Jhala D In-House Viral Transport Medium (VTM) manufacture in the time of shortage, supply and crisis of COVID-19 at Veteran Affairs Medical Center (VAMC). Am J Clin Pathol. 2020;154:S161. 10.1093/ajcp/aqaa161.352

[7] Pfau B Opsahl J Crew R Best S Han PD Heidl S Tiny swabs: nasal swabs integrated into tube caps facilitate large-scale self-collected SARS-CoV-2 testing. J Clin Microbiol. 2024;62:e0128523. 10.1128/jcm.01285-23 38131692 PMC10865831

[8] Saini V Kalra P Sharma M Rai C Saini V Gautam K A cold chain-independent specimen collection and transport medium improves diagnostic sensitivity and minimizes biosafety challenges of COVID-19 molecular diagnosis. Microbiol Spectr. 2021;9:e0110821. 10.1128/Spectrum.01108-21 34878310 PMC8653843

[9] Kashapov RN Tsibin A Comparison of the physical properties and effectiveness of medical swabs for sampling biomaterials. Biomed Eng (NY). 2021;55:289–93. 10.1007/s10527-021-10120-z 34776519 PMC8573295

[10] Vandenberg O Martiny D Rochas O van Belkum A Kozlakidis Z Considerations for diagnostic COVID-19 tests. Nat Rev Microbiol. 2021;19:171–83. 10.1038/s41579-020-00461-z 33057203 PMC7556561

[11] Gokulan CG Kiran U Kuncha SK Mishra RK Temporal stability and detection sensitivity of the dry swab-based diagnosis of SARS-CoV-2. J Biosci. 2021;46:95. 10.1007/s12038-021-00216-9 34728592 PMC8556569

[12] Khan MF Roopa C Dry Swab-Based Nucleic Acid Extraction vs. Spin Column-Based Nucleic Acid Extraction for COVID-19 RT-PCR Testing: A Comparative Study. Can J Infect Dis Med Microbiol. 2023;2023:6624932. 10.1155/2023/6624932 37663452 PMC10469701

[13] Naeem K Ilyas M Fatima U Nisar MI Kazi AM Jehan F Profile: Health and Demographic Surveillance System of Pakistan (KHDSS). Online J Public Health Inform. 2018;10:e160. 10.5210/ojphi.v10i1.8953

[14] Gallup N Pringle AM Oberloier S Tanikella NG Pearce JM Parametric nasopharyngeal swab for sampling COVID-19 and other respiratory viruses: Open source design, SLA 3-D printing and UV curing system. HardwareX. 2020;8:e00135. 10.1016/j.ohx.2020.e00135 32904317 PMC7455530

[15] Allana R Yildirim I Ariff S Belgaumi SM Ahsan N Aguolu O Determining the cause of death through mortality surveillance using verbal autopsy in Karachi, Pakistan. J Glob Health. 2025;15:04199. 10.7189/jogh.15.04199 40689473 PMC12278687

[16] Pillay S Giandhari J Tegally H Wilkinson E Chimukangara B Lessells R Whole genome sequencing of SARS-CoV-2: adapting Illumina protocols for quick and accurate outbreak investigation during a pandemic. Genes (Basel). 2020;11:949. 10.3390/genes11080949 32824573 PMC7464704

[17] Modi A Vai S Caramelli D Lari M The Illumina sequencing protocol and the NovaSeq 6000 system. Methods Mol Biol. 2021;2241:15–42. 10.1007/978-1-0716-1099-2_2 33961215

[18] Chan Zuckerberg IDHomepage. 2025. Available: https://czid.org. Accessed: 7 October 2025.

[19] Kilianski A Carcel P Yao S Roth P Schulte J Donarum GB Pathosphere. org: pathogen detection and characterization through a web-based, open source informatics platform. BMC Bioinformatics. 2015;16:416. 10.1186/s12859-015-0840-5 26714571 PMC4696252

[20] Padgett LR Kennington LA Ahls CL Samarasinghe DK Tu Y-P Wallander ML Polyester nasal swabs collected in a dry tube are a robust and inexpensive, minimal self-collection kit for SARS-CoV-2 testing. PLoS One. 2021;16:e0245423. 10.1371/journal.pone.0245423 33852576 PMC8046217

[21] Kritikos A Caruana G Brouillet R Miroz J-P Abed-Maillard S Stieger G Sensitivity of rapid antigen testing and RT-PCR performed on nasopharyngeal swabs versus saliva samples in COVID-19 hospitalized patients: results of a prospective comparative trial (RESTART). Microorganisms. 2021;9:1910. 10.3390/microorganisms9091910 34576805 PMC8464722

[22] Hart B Tu Y-P Jennings R Verma P Padgett LR Rains D A comparison of health care worker-collected foam and polyester nasal swabs in convalescent COVID-19 patients. PLoS One. 2020;15:e0241100. 10.1186/2052-0492-1-10 33108384 PMC7591034

[23] Codrington L Kuncio D Han J Nachamkin I Tolomeo P Hu B Yield of methicillin-resistant Staphylococcus aureus on moist swabs versus dry swabs. Am J Infect Control. 2013;41:469–70. 10.1016/j.ajic.2012.09.011 23337302

[24] Rose L Jensen B Peterson A Banerjee SN Arduino MJ Swab materials and Bacillus anthracis spore recovery from nonporous surfaces. Emerg Infect Dis. 2004;10:1023. 10.3201/eid1006.030716 15207053 PMC3323178

[25] Hedin G Rynbäck J Loré B New technique to take samples from environmental surfaces using flocked nylon swabs. J Hosp Infect. 2010;75:314–7. 10.1016/j.jhin.2010.02.027 20451296

[26] Donatelli J Macone A Goldmann D Poon R Hinberg I Nanji A Rapid detection of group A streptococci: comparative performance by nurses and laboratory technologists in pediatric satellite laboratories using three test kits. J Clin Microbiol. 1992;30:138–42. 10.1128/jcm.30.1.138-142.1992 1734045 PMC265009

[27] Bukhari AR Ashraf J Kanji A Rahman YA Trovão NS Thielen PM Sequential viral introductions and spread of BA. 1 across Pakistan provinces during the Omicron wave. BMC Genomics. 2023;24:432. 10.1186/s12864-023-09539-3 37532989 PMC10399012

[28] da Silva ACVF Silva CAO de Sousa GF Coelho VAGS da Cunha LT Paes AN Genomic surveillance and serological profile of SARS-CoV-2 variants circulating in Macaé and nearby cities, southeastern Brazil. Front Microbiol. 2024;15:1386271. 10.3389/fmicb.2024.1386271 38746751 PMC11091293

