# Supplementary material for: Validation of polyester nasal swabs for post-mortem SARS-CoV-2 diagnosis in Karachi, Pakistan: a prospective surveillance analysis
Source: J Glob Health. 2025 Nov 21;15:04288. doi: 10.7189/jogh.15.04288 (PMC12634021; doi:10.7189/jogh.15.04288)
Supplement: Online Supplementary Document [file jogh-15-04288-s001.pdf]

Supplement to: Allana R, Aziz F, Belgaumi SM, Kabir F, Yildirim I, Hotwani A, Malik FA, Aguolu O, Muneer S, Ahsan N, Hasan Z, Omer SB, Kazi AM. Validation of polyester nasal swabs for postmortem SARS-CoV-2 diagnosis in Karachi, Pakistan: a prospective surveillance analysis. J Glob Health, 2025;15:04288.

Supplementary Table S1: Comparison of Sequencing Quality and Genomic Coverage Between Dry and Wet Nasal Swab

**Parameters**	**Dry (n=19)**	**Wet (n=16)**
Average CT Value	29.1	31.3
%identification of Consensus Genome with the Reference Genome	99.7	99.6
Average Informative Bases/nucleotides	14,809	17,780
Average Missing Bases	10,627	9,977
Average Ambiguous Bases	0.89	2.8
Average Depth Coverage (x)	1215	1304
Average Breadth Coverage (%)	54.25	63.3
Average No. of SNPs	46.1	56

This table compares sequencing metrics of SARS-CoV-2 genomes from Dry and Wet swab storage conditions. Parameters include qRT-PCR CT values, genome coverage, depth, and SNP counts, obtained through standard NGS workflows.
